# Supplementary material for: Dynamics and Endocytosis of Flot1 in Arabidopsis Require CPI1 Function
Source: Int J Mol Sci. 2020 Feb 25;21(5):1552. doi: 10.3390/ijms21051552 (PMC7084554; doi:10.3390/ijms21051552)
Supplement: Supplementary file 1 [file ijms-21-01552-s001.pdf]

Supplemental Table 1. Primers used for PCR analysis of *Flot1* expression.

Experiment	Target gene	PCR product (bp)	Direction	Primer sequence (5′–3′)
RT-PCR/expression	Flot1	561	Forward	ATGAACGCTTTGACTCGAAC
			Reverse	TTAGCTGCGAGTCACTTGCTTCG
RT-PCR/expression	Actin	384	Forward	ATTCAGATGCCCAGAAGTCTTGT
			Reverse	GAAACATTTTCTGTGAACGATTCC
RT-PCR/expression	CPI	294	Forward	TGGGGAGAGCTCTTCTTCCT
			Reverse	ATAGGACGCTCCAAGAACTTT
PCR/expression	Flot1	1413	Forward	CGCGGATCCATGTTCAAAGTTGCAAG
			Reverse	CCGGAATTCTTAGCTGCGAGTCACTT
